# Machine Learning for Parkinson’s Disease Biomarkers: A Systematic Review of Diagnostic Performance, Risk of Bias, and Clinical Translation Readiness

**DOI:** 10.3390/brainsci16101027

**Published:** 2026-09-26

**Authors:** Osmar Pinto Neto, Tatiana Okubo Rocha Pinho

**Affiliations:** 1 Department of Kinesiology, California State University San Marcos (CSUSM), San Marcos, CA 92096, USA; pinho001@csusm.edu; 2 Neurometra, Carlsbad, CA 92009, USA; 3 Center of Innovation, Technology and Education (CITE) and Graduate Program in Biomedical Engineering, Anhembi Morumbi University, São José dos Campos 12247-016, SP, Brazil

**Keywords:** Parkinson’s disease, machine learning, deep learning, biomarkers, diagnosis, systematic review, risk of bias, external validation, PROBAST, clinical translation readiness

## Abstract

**Highlights:**

**What are the main findings?**

- Machine-learning studies for Parkinson’s disease consistently reported high diagnostic accuracy across very different biomarker modalities, but most models were evaluated only within their development datasets.
- Evidence for real-world generalizability was limited: risk of bias was common, external validation was rare, and practical deployment burden differed substantially across modalities.

**What are the implications of the main findings?**

- Future work should prioritize independent external validation, subject-independent testing, and complete reporting rather than further increases in already high internal accuracy.
- Lower-burden approaches such as voice, handwriting, gait, and wearable sensing may be attractive for scalable screening, whereas more resource-intensive modalities may be better suited to confirmatory or specialized clinical use.

**Abstract:**

Background/Objectives: Machine learning (ML) is frequently proposed for Parkinson’s disease (PD) diagnosis, but reported accuracy may not reflect methodological quality, generalizability, or clinical feasibility. We compared diagnostic performance, risk of bias, external validation, and deployment burden across biomarker modalities. Methods: We searched PubMed/Medical Literature Analysis and Retrieval System Online (MEDLINE, Web of Science, and Institute of Electrical and Electronics Engineers (IEEE) Xplore for studies from January 2020 through March 2026 using a Preferred Reporting Items for Systematic Reviews and Meta-Analyses (PRISMA) 2020-compliant protocol registered with the International Platform of Registered Systematic Review and Meta-analysis Protocols (INPLASY). AI-assisted screening was audited in 147 records (Cohen’s κ = 0.862). Reviewers verified extracted data and Risk Of Bias ASsessment Tool (PROBAST) judgments. Deployment burden was rated across five acquisition domains. Full-text assessment used a modality-stratified sample of retained records; we audited those not selected. Results: Of 8135 deduplicated records, 2046 were retained after screening and 377 reports were sampled for full-text assessment; the final synthesis included 264 studies across nine modalities. Among 252 studies reporting accuracy, median best-model accuracy was 95.3% (range 64.9–100%). However, under our adapted PROBAST rules, 259 studies (98.1%) were at high overall risk of bias; 215 (81.4%) remained at high overall risk when the external-validation item was excluded, and only 20 (7.6%) met the external-validation definition. In ten within-study comparisons, external performance fell in eight. Median deployment burden ranged from 1.0 (voice/speech) to 4.2 (neuroimaging). Conclusions: Reported performance has advanced faster than evidence of generalizability and clinical implementation. Clinical translation will require subject-independent evaluation, external multisite validation, and transparent reporting of performance and deployment requirements. These proportions describe the sample assessed, not the entire PD machine learning literature.

## 1. Introduction

### 1.1. The Diagnostic Challenge

Parkinson’s disease (PD) is the fastest-growing neurological disorder in terms of disability and death [1]. An estimated 8.5 million people live with PD worldwide [2], and the number of people living with the disease is projected to more than double by 2040 [3,4]. Pathologically, the disease is defined by progressive loss of dopaminergic neurons in the substantia nigra pars compacta, producing the cardinal motor signs of bradykinesia, rigidity, resting tremor, and postural instability [5,6]. Quantitative motor studies additionally demonstrate disease-related disturbances in grip-force control, tremor dynamics, bimanual coordination, and neuromuscular synchronization [7,8]. Non-motor features such as hyposmia, REM sleep behavior disorder, autonomic dysfunction, and cognitive change may precede motor onset by a decade or more [9].

Diagnosis continues to be essentially clinical, guided by the MDS Clinical Diagnostic Criteria [10]. Even in specialist hands, diagnostic accuracy reaches only about 80–84%, falling to roughly 73% in general neurology practice [11], and by the time motor symptoms prompt evaluation, an estimated 50–70% of nigrostriatal neurons have already degenerated [12]. This diagnostic uncertainty, together with the long period before clear motor symptoms emerge, may limit the potential benefit of disease-modifying therapies and motivates the search for objective, quantitative biomarkers that can identify PD earlier.

### 1.2. Machine Learning for Parkinson’s Disease Biomarkers

Machine learning (ML) and deep learning (DL) methods can identify subtle, high-dimensional patterns that are difficult to detect with conventional clinical assessment [13], and may support objective classification, earlier detection, integration of different data types, and low-cost data collection using non-invasive digital measures such as voice recordings and wearable sensors. Candidate digital biomarkers span multiple physiological domains: voice analysis has been used for data-driven PD classification [14], stabilometric measures can quantify postural abnormalities [15], and grip-force variability has been investigated as a motor biomarker for early detection [16]. Over the past several years, this literature has grown rapidly, driven by public datasets (e.g., the UCI Machine Learning Repository, PPMI, mPower, and PhysioNet), advances in model architecture (including convolutional and transformer networks and self-supervised learning) [17], and broader growth in digital health and precision neurology [18,19]. ML has been applied to voice and speech, gait and balance, neuroimaging, motor and handwriting tasks, electroencephalography, wearable-sensor signals, fluid and molecular assays, cognitive and clinical data, and multimodal combinations of these sources [20,21,22,23,24,25,26,27,28,29,30].

### 1.3. Rationale and Objectives

Despite this growth, clinical translation has been limited: few ML models have progressed to prospective clinical validation or routine practice. Existing reviews have often focused on a single modality [31,32] or summarized ML/DL methods without systematically evaluating evidence quality across modalities [33]. Two gaps are especially important. First, high accuracy is often reported without considering risk of bias, external validation, or subject-independent testing, making it difficult to know how well the models will perform in new clinical populations. Second, modalities are rarely compared on practical factors such as accessibility, cost, invasiveness, and scalability, even though these factors are essential for clinical use.

This systematic review addresses these gaps. Following a PRISMA 2020-compliant protocol registered with INPLASY, we synthesized a modality-stratified sample of 264 studies of ML-based PD biomarkers across nine modality categories. Our goals were to: (i) describe diagnostic performance within and across modalities; (ii) assess risk of bias using PROBAST, with emphasis on external validation and sample-size adequacy; and (iii) compare practical clinical-translation profiles across modalities by considering diagnostic performance and external-validation evidence alongside a study-derived deployment burden score covering five acquisition domains (Section 2.7). Compared with previous reviews, this approach combines cross-modality performance mapping, structured risk-of-bias assessment, and practical considerations for clinical use.

## 2. Materials and Methods

### 2.1. Protocol and Registration

This review was conducted and reported in accordance with the PRISMA 2020 statement [34] and the PRISMA-DTA extension for diagnostic accuracy studies [35]. The protocol was registered with the International Platform of Registered Systematic Review and Meta-analysis Protocols (INPLASY; registration number INPLASY202640002) on 1 April 2026; the protocol was received and published on that date, before the database searches were conducted on 2–4 May 2026, and specified the search strategy, eligibility criteria, data-extraction framework, risk-of-bias methodology, and planned synthesis. We provide the completed PRISMA 2020 checklist in Supplementary Table S1. We document any deviations from the registered protocol in Supplementary Note S1.

### 2.2. Eligibility Criteria

We defined eligibility using a Population, Index test, Comparator, Outcomes, and Study design (PICOS) framework adapted for diagnostic-accuracy studies. Population: adults (≥18 years) with PD diagnosed per established criteria (UK Parkinson’s Disease Society Brain Bank, Movement Disorder Society (MDS) Clinical Diagnostic Criteria, or equivalent; idiopathic PD intended where diagnostic criteria were reported), compared with healthy controls or patients with other neurological conditions; studies of atypical parkinsonism, drug-induced parkinsonism, or juvenile-onset PD alone were excluded. Index test: any supervised or semi-supervised ML/DL algorithm (e.g., support vector machines (SVMs), random forest, gradient boosting, logistic regression, k-nearest neighbors (k-NN), neural networks, convolutional neural networks (CNNs), recurrent neural networks (RNNs) and long short-term memory networks (LSTMs), transformers, autoencoders, ensembles) applied to human-derived biomarker data. Comparator (intended reference standard): clinical diagnosis by a movement-disorder specialist or neurologist using established criteria; self-reported diagnosis without clinical confirmation was not accepted. Outcomes: at least one of accuracy, area under the receiver operating characteristic curve (AUC/AUROC), sensitivity, specificity, or F1-score; studies reporting only regression outcomes (e.g., Unified Parkinson’s Disease Rating Scale (UPDRS)-score prediction) without a classification component were excluded. Study design: cross-sectional and longitudinal observational studies; randomized trials were eligible if they reported baseline ML classification prior to intervention. Diagnoses based solely on participant self-report without clinical confirmation were an exclusion criterion; however, widely used public datasets with incompletely documented reference standards (e.g., UCI or Kaggle) were retained when the report did not establish that PD status was based solely on self-report. The risk-of-bias assessment captured uncertainty in the reference standard rather than using it as grounds for exclusion.

Studies meeting any of the following were excluded: (E1) reviews, editorials, or commentaries without original data analysis; (E2) no ML/DL algorithm applied; (E3) focus solely on treatment-response or surgical-outcome prediction; (E4) animal or in vitro studies; (E5) total sample size below 20; (E6) conference abstracts without full text; (E7) no quantitative classification metric reported; (E8) non-English language; (E9) exclusively synthetic data; (E10) duplicate or incremental analysis of a previously included dataset without methodological novelty; (E11) no PD-versus-comparator diagnostic classification, that is, studies addressing severity, staging, progression or another within-PD task without distinguishing people with PD from a comparator group; (E12) eligibility could not be established because the report gave neither an interpretable participant-level sample size nor a diagnostic reference standard; (E13) publication date outside the stated window; and (E14) PD status established by participant self-report without clinical confirmation. We expanded the exclusion-code scheme to E1–E14 during full-text assessment; criteria E11–E14 captured additional observed reasons for ineligibility, and we recorded the expansion as a protocol deviation (Note S1). When a study reported multiple models on the same dataset, we selected the best-performing configuration as the primary result. Supplementary Table S3 lists the names of every machine learning method each study reported in the column “ML method(s) reported”, but we did not extract performance values for configurations we did not select; therefore, the extraction does not support re-analysis under an alternative model-selection rule. We recorded a study as externally validated only when the evaluation set came from a different cohort, site, or dataset than the one used for model development; a hold-out partition of the development dataset, however large, was not counted as external validation. We counted cross-dataset and leave-one-dataset-out evaluations as external. Where a study reported both an internal development design and an additional independent evaluation, the internal design is recorded in Supplementary Table S3 under “Primary validation design” and the independent evaluation is recorded separately under “External validation” and detailed in Table S7.

### 2.3. Information Sources and Search Strategy

We searched three bibliographic databases: PubMed/Medical Literature Analysis and Retrieval System Online (MEDLINE), Web of Science Core Collection, and Institute of Electrical and Electronics Engineers (IEEE) Xplore. The review team conducted searches manually from 2 to 4 May 2026 and covered publications from 1 January 2020 through 31 March 2026. For each database, we saved the exact query, result count, and record export in the project archive. We searched PubMed/MEDLINE and IEEE Xplore using disease-, machine learning-, and modality-specific terms across eight sub-searches. We searched the Web of Science Core Collection using a single combined disease-and-machine-learning query across all modalities. Supplementary Table S2 provides the complete search strings. Web of Science results were filtered to exclude Review Articles. We also searched Scopus, the ACM Digital Library, and Google Scholar but could not include these sources because of subscription limitations (Scopus and ACM) or automated query blocking (Google Scholar). Hand-searching the reference lists of included studies and key reviews added two records.

### 2.4. Study Selection

We deduplicated records programmatically by DOI, PubMed identifier, and normalized title, yielding 8135 unique records. Because the modality-stratified queries returned many off-topic records, we first applied a precision filter: we removed records with no mention of “Parkinson” in the title, abstract, keywords, or venue (*n* = 2550), leaving 5585 records for title-and-abstract screening.

Title-and-abstract screening was AI-assisted, using ASReview LAB (v3.0.5) in oracle mode with the ELAS u4 active-learning model (a support-vector classifier over TF-IDF features) [36]. Both reviewers screened records against the eligibility criteria: O.P.N. labeled 194 records, and T.O.R.P. labeled 280; both reviewers independently screened 147 records. Agreement on those 147 double-screened records was high (raw agreement 95.2%; Cohen’s κ = 0.862), and the seven disagreements were resolved by consensus. The pooled human labels trained a TF-IDF and logistic regression classifier that achieved a five-fold cross-validated AUC of 0.995 (SD 0.004). This classifier scored the remaining unlabeled records, and we carried forward those with a predicted relevance probability of 0.50 or above. We audited classifier behavior by manually reviewing a 90-record calibration sample spanning the decision boundary. A retraction audit against Crossref and PubMed matched 23 records. Sixteen of these were still in the relevant pool and were removed by the audit; the remaining seven had already been excluded by relevance screening, so no further action was needed. None of the 23 entered the retained set. Figure 1 counts all 23 among the records excluded at title-and-abstract screening. They precede the record-level ledger in Table S15, which begins at retrieval, so we list each with its screening decision in the screening data released with this review. This yielded 2046 records retained for potential full-text assessment.

We could not assess all 2046 records retained for potential full-text assessment within the review’s resources, so we drew a modality-stratified sample. Within each biomarker modality, we ranked records by citations per year, then by venue tier and publication year as tiebreakers, and retained the top 50; we carried modalities with fewer than 50 retained records forward in full and applied no cap to fluid-biomarker or microbiome records. Six modalities reached the cap of 50, and three had fewer eligible records (other wearable sensors, 40; unclassified at screening, 25; fluid and molecular biomarkers, 12); Supplementary Table S15 shows the sampling stratum for every record. We chose this purposive rather than random rule to concentrate limited full-text capacity on the most influential work in each modality. It biases the sample towards well-cited, higher-tier, and more recent studies. We did not test whether that selection was associated with lower risk of bias, so the direction of any resulting bias in the risk-of-bias findings is unknown. The rule produced 377 records, and hand-searching reference lists identified 2 additional reports. We sought full texts for all 379 reports and retrieved 378 (99.7%).

To test whether this selection distorted the review’s findings, we audited the 1669 retained records that were not selected. The audit could not use full texts, so it compared the 377 selected records with the 1669 unselected records on information available for every retained record: publication year, source database, venue type, the screening classifier’s relevance probability, the recorded abstract quality signals, and three quantities mined from the title, abstract, and keywords, namely, the highest accuracy value reported, whether an AUC was reported, and whether external-validation language was used. Two reconstructions were validated before use. A keyword-based rule correctly reproduced the recorded biomarker modality for 77.7% of the 377 selected records. The accuracy-mining rule identified an accuracy value for 57.8% of the included studies. Among those studies, the mined value was within 1 percentage point of the verified value reported in Table S3 in 80.0% of cases, with a median absolute difference of 0.00 percentage points. The audit could not directly compare risk-of-bias judgments, verified sample sizes, or verified performance measures because these variables require full-text review. Supplementary Table S16 reports all audit comparisons, and Supplementary Code S2 can reproduce them.

We extracted data using AI in two phases: regular-expression parsing followed by large-language-model extraction, which generated an initial automated pre-assessment of the PROBAST signaling answers for each report. We used the automated output only to organize the assessment workload; it did not affect eligibility. Human reviewers assessed all 378 full-text files obtained for the modality-stratified and hand-searched set against the eligibility criteria. One initially assessed file was later found not to correspond to the indexed report; because we could not obtain the correct full text, we excluded that report under E6. For all included studies, we verified the extracted fields and signaling answers against the corresponding source article. Screening-disagreement adjudication identified four additional records; we retrieved and assessed three under the same procedure but could not obtain one. We recorded reasons for full-text exclusion using the E1–E14 scheme and report them per record in Supplementary Table S15. The two reviewers verified disjoint sets of included studies, so no two-reviewer inter-rater statistic is available for this stage (see Limitations). We report concordance between the initial machine-generated risk-of-bias ratings and the final human-verified ratings in Supplementary Table S5, and per-record dispositions from retrieval onward in Supplementary Table S15.

### 2.5. Data Extraction

A standardized form (Microsoft Excel), pilot-tested on five studies, captured, for each study: identification (first author, year, journal, DOI, venue tier); sample sizes (total, PD group, control group) together with whether an interpretable subject-level count was available; the PD reference standard; biomarker modality; the task addressed; the data source, as a category and as the dataset names given in the report; the machine learning methods reported and the best-performing one; the validation design; performance metrics (accuracy, AUC, sensitivity, specificity, F1); and whether the model was evaluated on an independent external dataset. Every one of these fields is supplied for all included studies in Supplementary Table S3, from which Tables S8, S9 and S13 are computed. Several fields anticipated in the protocol were not captured as structured fields and are therefore not reported (Note S1): country, Hoehn and Yahr distribution, medication status, handcrafted versus learned features, hyperparameter tuning, confidence intervals, explainable-AI method, and code availability. Extraction was AI-assisted, and for every included study, one of two reviewers checked the AI-assisted extraction record against the corresponding source article. We divided included studies between the two reviewers rather than duplicating them, so each included study was verified once, as described in Section 2.4. We resolved queries raised during verification by consensus, and Supplementary Table S3 provides the complete extraction.

Because the reproducibility of a language-model-assisted review depends on details that are easy to omit, we state the procedure in full; Supplementary Table S21 provides the same information with the prompts verbatim, and we release the scripts used with the review. Two steps in the data-extraction and PROBAST pre-assessment pipeline used a language model, and both used the same one: gemma4:26b, an open-weight model served locally through Ollama, with temperature 0.1 and JSON-mode output. The first step, phase 2 of data extraction, returned the extraction fields for one study per call; phase 1 was regular-expression parsing with no model involved. The second step returned all 22 PROBAST signaling answers for one study per call. The model was not given complete articles. For each study, text was extracted from the first 25 PDF pages with pypdf, sliced from the first Methods heading to the first Discussion, Conclusion, References, or Acknowledgments heading, and truncated to 6000 characters for extraction and 8000 for the risk-of-bias step; where that slice came out shorter than 1500 characters, the script used the opening characters of the whole text instead. Results tables therefore usually fell outside the excerpt, which is a real limitation of the pre-assessment and part of why human verification changed as much as it did. We processed each study once and did not repeat runs or measure run-to-run consistency. We counted a study as a failure if its PDF was missing or unreadable and left it blank rather than guessing; we also left blank any response that did not parse as JSON, and the human reviewer completed those rows. The model also returned its own domain and overall risk-of-bias ratings, which we discarded; we recomputed every rating reported here from the verified signaling answers using the rule in Table S4. One of the two reviewers then opened the source article for every one of the 264 included studies and checked the extracted fields and every signaling answer against it. Table S5 reports how far the pre-assessment and the verified ratings diverged, which was substantial, and Table S19 reports agreement between the two reviewers on a 31-study subset.

In 46 studies, the report provided only recording, image, or signal-level counts, with no interpretable subject-level sample size. We did not contact authors to obtain missing counts. We retained these studies in the performance synthesis when they reported a quantitative classification metric but excluded them from subject-level sample-size analyses and flagged them in the “Subject-level *n* interpretable” column of Table S3. Two further studies reported an interpretable participant total but did not give a fully interpretable participant-level group split; we retained their totals and did not use their group counts. Table S13 therefore counts 218 studies with an interpretable subject-level sample size.

### 2.6. Risk-of-Bias Assessment

We assessed risk of bias using PROBAST (Prediction model Risk Of Bias ASsessment Tool), which was developed for diagnostic and prognostic prediction models. It evaluates four domains (participants, predictors, outcome, and analysis) using structured signaling questions. The analysis domain included ML-specific issues such as subject-independent validation, use of an external test set, sample-size adequacy, information leakage during tuning of hyperparameters or feature selection (for example, when these steps were performed before data partitioning or outside the training folds), and handling of class imbalance. Each signaling question was rated yes, probably yes, probably no, no, or no information; a domain was rated high risk if any signaling question indicated risk, unclear if information was insufficient, and low otherwise, with overall risk derived accordingly. Because absence of an external test set is one of the machine learning signaling questions in the analysis domain, a study with no external evaluation is rated high risk on that item alone. We therefore report two quantities separately throughout: methodological risk of bias, with a sensitivity analysis that excludes the external-validation item, and evidence of external generalizability, expressed as the proportion of studies with an independent external evaluation. The complete set of signaling questions is provided in Supplementary Table S4. PROBAST asks whether there was a reasonable number of participants with the outcome and sets no numerical threshold. The required size depends on the number of candidate predictor parameters, the outcome proportion, and the expected predictive performance rather than on any fixed events-per-variable rule [37], and conventional cross-validation biases machine learning performance estimates from small samples [38]. During revision, we therefore replaced the numeric bands used at submission with an explicit decision rule and froze the rule before the recheck. For all 264 included studies, we recorded the inputs the rule consumed: the interpretable subject-level count, the size of the smaller diagnostic group, the number of features or predictors entering the final model where stated, and a complexity class. We initially extracted these inputs automatically from the source PDFs, with targeted human rereading as described below. The complexity class is C1 when the final model is fitted to a countable feature set, including a classical learner or a shallow network applied to handcrafted features or to frozen pretrained embeddings; C2 when an end-to-end deep architecture is fitted to raw or minimally processed signals, images or spectrograms; and C3 when the report does not describe the model well enough to place it in either. The rule is applied in order: no information when no interpretable subject-level count is available; no when the subject-level sample is below 50, or the smaller diagnostic group contains fewer than 20 participants; no information for class C3; for class C1, no when a stated feature count leaves fewer than 10 participants per feature, otherwise probably yes between 50 and 99 participants and yes at 100 or more; and for class C2, probably yes at 500 participants or more and probably no below that. The 10-participant-per-feature screen identifies clear inadequacy rather than sufficiency, and the 500-participant boundary for end-to-end models is a review-specific operational choice reflecting that these architectures carry parameter counts orders of magnitude above the participant counts in this literature; we repeated the re-rating with boundaries of 250 and 1000 participants. Supplementary Table S6 gives the rule-based answer, the answer recorded before revision, and the recorded inputs for every study. Note S1 records the change as a protocol deviation, and Supplementary Code S3 reproduces the rule. Both reviewers then independently rated a stratified subset of 31 studies against the frozen rule, without seeing each other’s answers or the rule-based ratings, reaching 93.5% raw agreement on the item answer (Cohen’s κ = 0.91; prevalence-adjusted bias-adjusted κ = 0.87). Each reviewer agreed with the rule-based recheck less often (64.5% and 71.0% of that subset) because the recheck read text extracted automatically from the source PDFs and more often recorded a participant count or group size the article did not support, in a direction that favored judging samples adequate. We therefore identified the 71 studies whose answer would change if any single recorded input were wrong, divided them between the two reviewers, and re-read each against the source article; that pass changed 29 of the 71 answers, 27 of them to a stricter rating. Of the 264 studies, 103 were read by a reviewer against the frozen rule and the remaining 161 were rechecked using the rule-based method; Table S6 records which. Risk-of-bias verification was divided between the two reviewers rather than duplicated, so each included study carries a single human rating across the corpus. To measure how reproducible those judgments were, both reviewers then rated the same 31 studies independently and blind to each other, the recorded ratings, and the automated pre-assessment. The 31 were drawn by proportional allocation throughout the nine modalities, and the 14 signaling questions covered domains 1 to 3 in full together with the five analysis-domain items that reproduced the full 13-item domain rating for all 264 included studies, so every domain rating and the overall rating can be recomputed from each reviewer’s answers. Agreement is reported in Section 3.2 and per item in Table S19. For the corpus as a whole, we do not report a two-reviewer comparison. Supplementary Table S5 instead reports concordance between the initial automated ratings and the final human-verified ratings. This comparison was possible for 257 of the 264 included studies: agreement was 61.9% for domain 1, 70.4% for domain 2, 89.9% for domain 3, and 45.1% for domain 4, with agreement on the overall rating in 45.1% of studies and on 64.1% of individual signaling answers. Seven included studies lacked a directly comparable automated pre-assessment: three had no automated pre-assessment, and four required complete assessment from the correct report because the linked full text did not correspond to the indexed article. The lower concordance in the analysis domain was concentrated in the external-validation and sample-size items.

In a separate 31-study dual-rating exercise, both reviewers rated the same 31 studies independently, blind to each other and to the recorded ratings, on 14 signaling questions covering domains 1 to 3 in full and the five analysis-domain items that reproduced the full domain rating for all 264 included studies (Section 2.6). The overall rating came out the same for 30 of the 31 studies (96.8%; prevalence-adjusted bias-adjusted κ = 0.94), and the analysis domain for 29 (93.5%; PABAK = 0.87); for these two, almost every study falls in one category, so Cohen’s κ is uninformative, and the prevalence-adjusted figure is the one to read. Agreement was 83.9% for the outcome domain (κ = 0.70), 77.4% for participant selection (κ = 0.38), and 74.2% for the predictor domain (κ = 0.47). Agreement on the individual signaling answers was lower, 52.8% across all five response options (κ = 0.37), and the reason is visible in the answers: 88 of the 205 disagreements are between “no” and “probably no”, or between “yes” and “probably yes”, on the same evidence, which PROBAST aggregates identically and which therefore cannot change any rating. Scored on the three categories the tool actually aggregates, agreement rises to 73.0% (κ = 0.57), and on the single question of whether an item indicates risk at all to 85.3% (κ = 0.60). Among the remaining disagreements, many involved how to read silence, that is, whether a method an article does not describe should be recorded as not done or as no information; these were concentrated in the participant domain and were the disagreements most able to move a rating. Table S19 reports every figure per item and per domain. We therefore regard the overall high-risk classification as reproducible between reviewers, while the individual signaling answers behind it are not interchangeable.

### 2.7. Data Synthesis

Because modalities, algorithms, datasets, and reporting varied substantially, we used a narrative synthesis organized by modality. Within each modality, we summarized sample characteristics, feature extraction, algorithms, validation strategies, and classification performance. Cross-modality comparisons were descriptive because differences among datasets, preprocessing, and validation did not support formal statistical pooling; therefore, we did not perform a meta-analysis. We also evaluated practical considerations relevant to clinical translation. The registered protocol specified consideration of practical translation criteria but did not define a numerical deployment-burden index. Before final synthesis, we operationalized these criteria as a review-specific five-domain rubric applied at the study level. For each included study, we rated acquisition cost, participant burden/invasiveness, equipment and infrastructure requirements, scalability/throughput, and feasibility of remote collection from 1 (lowest burden) to 5 (highest burden) using predefined anchors. We calculated the unweighted mean of the five ratings as the study-level deployment-burden score. We summarized modality-level burden using the median study-level score and interquartile range; we examined means as a sensitivity analysis. The burden index measures practical deployment burden only and does not incorporate diagnostic performance, sample size, external validation, or risk of bias. We used large-language-model assistance to locate acquisition descriptions in the source articles and generate initial ratings according to the predefined rubric; the authors reviewed the resulting ratings and source-based rationales before finalization. This use is also reported in the Declaration of Generative AI and AI-Assisted Technologies. Supplementary Table S13 provides the scoring anchors, decision rules, modality summaries, and sensitivity analyses, and Table S14 provides all 264 study-level ratings and source-based rationales. We treated AUC/AUROC as a measure of discrimination, with 0.5 indicating chance and 1.0 indicating perfect discrimination. Where a study reported a metric only as an inequality (for example, an AUC given as “>0.98”), we treated it as not reporting a point estimate and excluded it from the corresponding median; this affected one study.

## 3. Results

### 3.1. Study Selection

Database searching returned 10,828 records (PubMed, *n* = 1572; IEEE Xplore, *n* = 3468; Web of Science, *n* = 5788). After removing 2693 duplicates, 8135 unique records remained. A precision filter removed 2550 records with no mention of Parkinson’s disease in the title, abstract, keywords, or venue, leaving 5585 records for title-and-abstract screening, of which 3539 were excluded. Of the 2046 records retained for potential full-text assessment, 377 were selected by modality-stratified sampling, and two additional reports were identified by hand-searching reference lists. We sought full texts for 379 reports and obtained 378, all of which underwent initial eligibility assessment. Eighty-six were excluded at full-text assessment, most commonly because the study addressed a within-PD task rather than PD-versus-comparator classification (E11, *n* = 52) or because eligibility could not be established from the report (E12, *n* = 13); the full breakdown is given in Figure 1 and per record in Table S15. We later found one initially assessed file did not correspond to the indexed report; we excluded it because we could not obtain the full text. Resolving the seven title-and-abstract screening disagreements identified four relevant records; we retrieved and assessed three and could not obtain one. Eligibility confirmation against the prespecified date window and diagnostic-task criteria excluded 30 studies. Seventeen did not contain a PD-versus-comparator diagnostic classification used in this review (E11, *n* = 14; E3, *n* = 2; E9, *n* = 1), twelve fell outside the stated publication window, and one determined PD status by participant self-report. The final synthesis included 264 studies (Figure 1).

The 1669 records not selected for full-text assessment differed from the 377 selected records as intended by the sampling rule (Table S16). Selected records were more recent (median publication year 2025 vs. 2024), more often published in journals rather than conference proceedings (62.9% vs. 39.8%), and more completely reported in the abstract: a performance metric appeared in 88.9% vs. 79.3%, a sample size in 36.9% vs. 23.4%, and an AUC in 16.4% vs. 10.2%. The two characteristics most relevant to this review’s conclusions differed little. The highest accuracy reported in the abstract had a median of 96.7% among selected records and 95.8% among unselected records, a difference of 0.9 percentage points (bootstrap 95% CI −0.4 to 1.9; *p* = 0.21); restricting both groups to records whose abstracts carried both a machine learning cue and a PD-versus-comparator cue gave a difference of 0.4 percentage points (95% CI −1.6 to 1.6; *p* = 0.90). External-validation language appeared in 2.7% of selected and 2.5% of unselected abstracts (*p* = 0.88), although this cue identified only five of the 20 studies we judged externally validated from the full text; therefore, it can rule out only a large difference. Selection therefore favored more recent, journal-published and more completely reported work without detectably shifting reported accuracy, while leaving the representativeness of the risk-of-bias estimates untested.

### 3.2. Risk of Bias

We assessed risk of bias for all 264 included studies across four PROBAST domains (Table 1; full per-study ratings in Supplementary Table S6). Under the adapted rules described in Section 2.6, 259 studies (98.1%) were judged at high risk of bias and the remaining five (1.9%) at unclear risk, with no study at low risk; because absence of an external test set is one of the analysis-domain signaling questions, this overall rating combines methodological limitations with absence of evidence for generalizability, which we separate below. This classification was driven by the analysis domain, which was high risk in 259 studies (98.1%) and unclear in the remaining five (1.9%).

The most common weakness was the lack of external validation. Only 20 of 264 studies (7.6%) evaluated their models on an independent external cohort or dataset. In contrast, 218 (82.6%) used only internal validation, and 26 (9.8%) did not describe validation clearly enough to judge. Small sample sizes added to this concern: under the rule described in Section 2.6, 162 studies (61.4%) had a sample judged inadequate for the model fitted and a further 57 (21.6%) could not be judged because no interpretable subject-level count was reported. The rule-based answers agree with the judgments recorded before revision for 146 of the 264 studies (55.3%). Among the 118 exact-answer differences, 88 moved to a stricter aggregate risk category, 25 changed within the same aggregate category, and five moved to a less strict category. The largest single group of exact-answer changes, 61 of 118, comprised end-to-end deep models with fewer than 500 participants, which the rule rates as probably inadequate and which had often been rated adequate on participant count alone. With the end-to-end boundary at 250 or at 1000 participants, the number judged inadequate was 155 and 167, respectively (Table S6). Among the 218 studies reporting an interpretable subject-level count, the median total sample size was 105 participants (range 24–33,980); the remaining 46 studies reported only recording-, image-, or signal-level counts.

The 20 externally validated studies differed in design and strength of evidence (Table S7): one used a cross-site subset within the same consortium; twelve used an independent cohort or dataset with distinct acquisition; six used a leave-one-site, group, language, corpus, or dataset-out design across independent datasets; and one used a cross-disorder transfer design. Several external test sets were small and came from single institutions, so larger multisite confirmation is still needed. Because only 20 studies met the definition and only ten of those permitted a direct internal-to-external comparison, and because those twenty differed in design, cohort, and modality, everything reported about them here remains descriptive and should not be read as an estimate of the expected performance drop in general. For two of the leave-one-group/language-out studies, the verifying reviewer graded the external-validation item “probably yes” rather than “yes”, judging the cross-corpus partition not to constitute a fully independent external clinical cohort; both are retained under the prespecified design definition above, and both showed a substantial drop on the held-out group (Table S7). Under the stricter reading, 18 studies (6.8%) rather than 20 (7.6%) would be counted as externally validated.

The other PROBAST domains were more favorable but still imperfect. Selection of participants was rated low risk in 151 studies (57.2%), unclear in 65 (24.6%), and high in 48 (18.2%). Unclear ratings dominated the predictor and outcome domains (158 [59.8%] and 215 [81.4%], respectively), reflecting incomplete reporting rather than demonstrably flawed methods. The PD reference standard was established using explicitly named criteria in 45 studies (17.0%); a further 58 (22.0%) described a clinical diagnosis without naming the criteria, and 161 (61.0%) did not state how the diagnosis was made. Studies that did not state the criteria frequently drew on public repositories such as the UCI or Kaggle datasets, which do not document how the PD label was established. Collectively, results show that the high accuracies prevalent in this literature should be interpreted with caution, as internal-only validation risks optimistic estimates that may not generalize to independent clinical populations.

Because the absence of external validation is one of the machine learning-specific signaling questions in the analysis domain, and because a domain is rated high risk if any signaling question indicates risk, this single item strongly influences the overall judgment. In a sensitivity analysis excluding the external-validation item from the analysis-domain rule and recomputing overall risk across all four domains, 215 studies (81.4%) remained at high risk of bias, and 49 (18.6%) were rated unclear, with no study at low risk, compared with 259 (98.1%) and five (1.9%) under the primary scoring. The sample-size item has a smaller influence on the overall judgment: excluding it instead leaves 236 studies (89.4%) at high risk, 27 (10.2%) unclear and one (0.4%) at low risk, and excluding both items leaves 119 (45.1%) at high risk, 144 (54.5%) unclear and one (0.4%) at low risk, so the overall rating depends on the sample-size operationalization for 23 of the 264 studies. This is also where the adapted framework used here differs most from PROBAST+AI, which assigns external evaluation to a separate model-evaluation part (Table S11). The overall risk-of-bias assessment therefore reflects two separate contributions that should be read separately: methodological and reporting limitations, and the near-universal absence of external validation, which limits evidence of generalizability.

### 3.3. Diagnostic Performance by Biomarker Modality

The 264 included studies covered nine biomarker categories (Table 2). Throughout this section, we count a named benchmark by the number of studies that name it in Table S3; a study may name more than one, and private-cohort counts come from the single data-source category recorded for each study. Neuroimaging (*n* = 48), voice and speech (*n* = 44), motor and handwriting analysis (*n* = 40), EEG (*n* = 44), and gait and balance (*n* = 40) together accounted for 216 studies (81.8%). The remaining studies examined multimodal fusion (*n* = 22), other wearable-sensor approaches (*n* = 16), fluid and molecular biomarkers (*n* = 7), and cognitive or clinical data (*n* = 3). One cognitive/clinical study used nationwide health-screening data [39]. Reported classification performance was high across modalities. Among the 252 studies reporting accuracy, the median best-model classification accuracy was 95.3% (range 64.9–100%), and modality medians ranged from 91.2% to 97.1%. Interpret these values alongside the risk-of-bias findings (Section 3.2): most accuracies came from internal validation based on small, often public datasets, and only 20 studies (7.6%) used an independent external cohort. Unless stated otherwise, reported metrics refer to each study’s best-performing model in its primary PD-classification task.

Because modality medians combine different classification tasks, we also grouped studies by the task each addressed (Table S8). Diagnostic or detection studies (*n* = 251) had a median best accuracy of 95.5% across the 240 reporting one, while the smaller groups addressing severity or staging (*n* = 6) and progression or prediction (*n* = 3) had medians of 94.4% and 90.7% across six and two studies, respectively. Four studies were categorized as “other or not stated” and had a median accuracy of 94.3%. The review did not record the number of outcome classes per study, so it does not distinguish binary from multi-class diagnostic tasks, and the four task categories above are too coarse to remove that heterogeneity. We tested whether the distinction could be recovered from the reports themselves and found that it could not: explicit binary-task language appeared in 118 of the 264 full texts and explicit multi-class language in 87, but 54 used both vocabularies and 113 used neither, so at most 97 reports could be classified this way, and even those rested on a phrase that may belong to a related-work paragraph rather than to the study’s own design (Table S18). The medians reported here therefore pool classification problems of different difficulty, from PD against healthy controls to differential diagnosis and multi-class staging, and modality medians should be compared with that in mind rather than read as estimates of the same quantity.

#### 3.3.1. Neuroimaging (*n* = 48)

Neuroimaging was the largest category, dominated by structural MRI and dopamine-transporter (DaTSCAN) SPECT, with PPMI named in 28 studies, more than any other dataset, alongside six studies on in-house cohorts. Methods were almost exclusively deep learning: convolutional neural networks, transfer-learning backbones (ResNet, DenseNet, EfficientNet), and capsule networks, often with ensembling. The median accuracy was 97.1% (range 65–100%, k = 46), and the median AUC was 0.96 across 23 studies. This was the highest median among modalities with at least four accuracy-reporting studies. The highest reported figures in this modality were 100%, 100%, and 99.9%. Two studies in this modality reported exactly 100% accuracy, and seven reported 99% or above. Other results above 98% included a 2D hybrid capsule network distinguishing early PD from controls on PPMI (99.1%), AdaBoost on PPMI features (98.9%), and several three-class Alzheimer/PD/healthy classifiers on public Kaggle MRI sets reaching 98.5–99.1%. These last results warrant skepticism: the provenance and labeling of these public “PD MRI” collections are poorly documented, and >98% three-way structural-MRI separation of Alzheimer’s disease, PD, and healthy controls generates considerable concern about dataset artifacts, confounding, or information leakage as opposed to genuine discriminative signal. These values warrant caution: many MRI studies are trained on image- or slice-level samples without subject-independent partitioning, and a methodological critique showed that slice-level pooling inflated accuracy by roughly 26 percentage points, with subject-level accuracy falling to about 67%. Only four studies were validated externally, and 47 of the 48 studies were at high risk of bias. All 48 included neuroimaging studies are detailed in Supplementary Table S3 [27,40,41,42,43,44,45,46,47,48,49,50,51,52,53,54,55,56,57,58,59,60,61,62,63,64,65,66,67,68,69,70,71,72,73,74,75,76,77,78,79,80,81,82,83,84,85,86].

#### 3.3.2. Electroencephalography (*n* = 44)

EEG studies used resting-state and task-evoked recordings, typically from small single-site cohorts (the UC San Diego, University of New Mexico, and Iowa datasets recurred most often, named in nine studies between them, frequently with fewer than 50 participants). Counting each study’s best-performing model, convolutional networks led (16 studies), supplemented by SVMs (six) and hybrid CNN–transformer or CNN–LSTM models. EEG showed a median accuracy of 96.6% (range 75–100%, k = 44), with a median AUC of 0.98 across 18 studies. The highest reported figures in this modality were 100%, 100%, and 100%; three studies reported exactly 100% accuracy, and twelve reported 99% or above. Other high-performing results included a collaborative-representation classifier reaching 100% on a 60-subject stimulus-evoked dataset, a tunable-Q wavelet deep residual shrinkage network at 99.9% on sleep EEG, and an SVM with fuzzy-entropy features at 99.9%. A convolutional–transformer ensemble and a cross-dataset study spanning the UC San Diego, UNM, and Iowa cohorts were among the four studies that assessed across independent datasets. The combination of near-perfect accuracies and very small samples strongly indicates overfitting risk; only four studies validated externally, and all 44 studies were at high risk of bias. All 44 included EEG studies are detailed in Supplementary Table S3 [23,87,88,89,90,91,92,93,94,95,96,97,98,99,100,101,102,103,104,105,106,107,108,109,110,111,112,113,114,115,116,117,118,119,120,121,122,123,124,125,126,127,128,129].

#### 3.3.3. Motor and Handwriting (*n* = 40)

This category covered digitized handwriting and drawing (spiral, meander, figure-copy), finger-tapping, grip force, and tremor kinematics, predominantly using private datasets (11 studies) supplemented by the PaHaW/NewHandPD/HandPD family, named in seven studies. SVMs, convolutional networks (including ResNet and Vision Transformer architectures), and task-level ensembles were most common. The median accuracy was 96.0% (range 78–100%, k = 36), and the median AUC was 0.96 across 12 studies. The highest reported figures in this modality were 100%, 100%, and 100%; three studies reported exactly 100% accuracy, and seven reported 99% or above. Other handwriting studies also approached perfect classification: logistic-regression/CART models achieved 100% on selected tasks, a task ensemble reached 99.98% on PaHaW, and a Vision Transformer pipeline reached 99.9%. A minority used control-inclusive staging or multiclass tasks, or PD-versus-essential-tremor discrimination, rather than simple binary PD-versus-healthy-control classification. Small per-task sample sizes and heavy image augmentation likely contributed to the very high reported accuracies. Two of the 40 studies were validated externally; one did so via a leave-one-language-out design across multilingual handwriting corpora, and 39 of the 40 studies were at high risk of bias; that externally validated study is the exception, and it is rated unclear rather than low. All 40 included motor and handwriting studies are detailed in Supplementary Table S3 [22,130,131,132,133,134,135,136,137,138,139,140,141,142,143,144,145,146,147,148,149,150,151,152,153,154,155,156,157,158,159,160,161,162,163,164,165,166,167,168].

#### 3.3.4. Gait and Balance (*n* = 40)

Gait and balance studies drew on wearable inertial sensors, instrumented force platforms, posturography, and markerless video pose estimation, with 21 studies citing the PhysioNet gait database and 16 drawing on in-house cohorts. Counting each study’s best-performing model, SVMs (eight studies) and random forests (seven) were most frequent, alongside gradient boosting and CNN–RNN hybrids. Median accuracy was 96.1% (range 78–100%, k = 40), and median AUC was 0.97 across 13 studies. The highest reported figures in this modality were 100%, 99.8%, and 99.6%; one study reported exactly 100% accuracy, and seven reported 99% or above. Several top results approached 100% accuracy but were either multi-disease classifications or lacked subject-independent evaluation. For example, an SVM ensemble reporting 100% on PhysioNet was evaluated on inputs overlapping its training data, and a possibility-based decision tree reached 99.8% on the public PhysioNet gait database. The recurrent use of the PhysioNet database without subject-level splits is a recognized source of optimistic bias. Only two studies were validated externally, and all 40 studies were at high risk of bias. All 40 included gait and balance studies are detailed in Supplementary Table S3 [25,169,170,171,172,173,174,175,176,177,178,179,180,181,182,183,184,185,186,187,188,189,190,191,192,193,194,195,196,197,198,199,200,201,202,203,204,205,206,207].

#### 3.3.5. Voice and Speech (*n* = 44)

Voice and speech studies analyzed sustained phonation and connected speech, depending heavily on a small number of public benchmarks; the UCI voice collections appeared in 17 studies and were supplemented by in-house recordings (five). Classical models with feature selection (SVM, KNN) remained common, increasingly joined by CNNs and self-supervised speech embeddings (wav2vec, WavLM). The median accuracy was 95.0% (range 76–100%, k = 43), with a median AUC of 0.97 across 15 studies. Four studies in this modality reported 100% accuracy, and six reported 99% or above. Other strong results included logistic regression with genetic-algorithm feature selection at 100% on the Italian dataset (90% on the KCL dataset), an SVM at 98.7%, and a KNN at 97.4% on UCI Oxford. Because a handful of small datasets are reused across many studies, apparent reproducibility may overstate independence; encouragingly, five studies validated across cohorts, including a WavLM-to-ImageBind fusion tested on external clinical samples and leave-group-out evaluation covering Italian and English corpora. With 43 of 44 studies at high risk of bias, the dominant limitation—internal validation on a few shared benchmarks—persisted. All 44 included voice and speech studies are detailed in Supplementary Table S3 [208,209,210,211,212,213,214,215,216,217,218,219,220,221,222,223,224,225,226,227,228,229,230,231,232,233,234,235,236,237,238,239,240,241,242,243,244,245,246,247,248,249,250,251].

#### 3.3.6. Multimodal Fusion (*n* = 22)

Multimodal studies combined two or more data types (most often neuroimaging with clinical variables, or voice with gait and tapping) and used private datasets (four studies), with PPMI named in eight. Approaches ranged from classical voting ensembles to deep cross-attention and feature-fusion networks. Median accuracy was 93.0% (range 80–100%, k = 21), and median AUC was 0.96 across 13 studies. The highest reported figures in this modality were 100%, 99.5%, and 98.8%; one study reported exactly 100% accuracy, and two reported 99% or above. Other high-performing results included a cross-attention CNN at 97.5% for a three-class prodromal/PD/control task on PPMI that also validated externally on the independent OASIS-3 and PDBP cohorts (96.2% and 95.8%), and a MobileNet–ShuffleNet fusion network at 95.6%. Fusion did not consistently exceed the best single-modality models, and the added complexity was rarely accompanied by stronger validation: only two studies were externally validated, and 21 of the 22 studies were at high risk of bias. All 22 included multimodal fusion studies are detailed in Supplementary Table S3 [24,30,252,253,254,255,256,257,258,259,260,261,262,263,264,265,266,267,268,269,270,271].

#### 3.3.7. Other Wearable Sensors (*n* = 16)

Beyond gait-specific applications, wearable-sensor studies used inertial measurement units, accelerometers, smartwatches, and single-lead ECG to capture general movement, tremor, or autonomic signals, overwhelmingly on private cohorts (nine studies) with a few public sets (PD-BioStampRC21, PADS). Counting each study’s best-performing model, SVMs (four studies) and random forests (three) were most common. The median accuracy was 91.2% (range 74–100%, k = 15), and the median AUC was 0.93 across five studies, including several perfect or near-perfect results on small private datasets: SVM and random forest models at 100% and XGBoost at 99.6%. The highest reported figures in this modality were 100%, 99.6%, and 96.2%; one study reported exactly 100% accuracy, and two reported 99% or above. As with other sensor modalities, small private cohorts and the absence of any external validation make these figures difficult to generalize; all 16 studies were at high risk of bias. All 16 included wearable-sensor studies are detailed in Supplementary Table S3 [29,272,273,274,275,276,277,278,279,280,281,282,283,284,285,286].

#### 3.3.8. Fluid and Molecular Biomarkers (*n* = 7)

Fluid-biomarker studies applied ML to blood and serum, cerebrospinal fluid, urine, and multi-omics or gene-expression data, using PPMI and in-house cohorts. Random forests and feed-forward neural networks predominated. The median accuracy was 94.1% (range 87–100%, k = 5), and the median AUC was 0.95 across five studies. The highest reported figures in this modality were 100%, 96.5%, and 94.1%; one study reported exactly 100% accuracy. A feed-forward network reached 96.5% on PPMI, but it combined multimodal inputs (cerebrospinal-fluid markers, SPECT imaging, and premotor features such as olfaction and REM-sleep behavior) rather than fluid markers alone, and a Rotation Forest classifier achieved 94.1% on a Brazilian serum cohort. Its “external” test, however, was a hold-out split of that single cohort rather than an independent dataset. The one genuinely external study in this category was informative: a random forest model’s AUC fell from 0.83 on internal cross-validation to 0.72 on an external University of Pennsylvania cohort, showing how internal validation can overestimate performance. Six of the seven studies were at high risk of bias. All seven included fluid and molecular biomarker studies are detailed in Supplementary Table S3 [21,287,288,289,290,291,292].

#### 3.3.9. Cognitive and Clinical Data (*n* = 3)

The smallest category comprised three studies using cognitive test scores, clinical and demographic variables, longitudinal clinical data, or nationwide health-screening measures. Two studies reported accuracy; their median was 95.6%, which should not be over-interpreted from so few studies. An AdaBoost ensemble classified mild cognitive impairment, PD, and Alzheimer’s disease from cognitive scores at 96.2% (AUC 0.97), and a random forest model on routinely collected patient data reached 95.0%. The third study used nationwide health-screening variables to predict PD risk and reported AUC rather than accuracy. None was externally validated, and all three were rated at high risk of bias. The narrow size of this category reflects the eligibility requirement that the model distinguish PD from a comparator rather than predict only a within-PD outcome such as fall risk, cognitive decline, or treatment response. No firm conclusion about cognitive and clinical data as a biomarker modality should be drawn from only three studies. All three included cognitive and clinical studies are detailed in Supplementary Table S3 [39,293,294].

#### 3.3.10. Cross-Modality Synthesis

Across the nine modalities, median reported accuracy varied little, from 91.2% to 97.1%. Interpret these differences cautiously because most studies relied on internal validation, several public datasets were repeatedly reused, and some studies did not clearly separate participants between training and testing. Only 20 studies included external validation, and performance was not consistently maintained in independent data (Table 3).

In contrast to accuracy, deployment burden differed considerably across modalities. Voice/speech had the lowest median burden (1.0), followed by cognitive/clinical (2.0; *n* = 3) and motor/handwriting (2.2). Gait/balance, other wearable sensors, and multimodal/fusion each had a median burden of 2.6. Multimodal/fusion, however, showed a wide range of burden scores (IQR 1.6–4.2), indicating that studies combined very different data types. EEG had a median burden of 3.4, fluid/molecular biomarkers 4.0, and neuroimaging 4.2. On acquisition burden alone, voice and wearable approaches are the least demanding and imaging and laboratory biomarkers the most, which bears on where each might practically sit in a pathway but not on whether any of them performs well enough to occupy it. Burden and translation readiness are different quantities, and Section 4.5 keeps them apart. Both reviewers scored the five rubric domains independently for the 31 studies used in the risk-of-bias dual rating: their scores agreed exactly for 84.5% of the 155 paired domain scores and to within one point for all of them, with a linearly weighted κ of 0.90 and an intraclass correlation for a single rater of 0.96 (0.99 for the composite), and they placed 28 of the 31 studies in the identical descriptive burden band. Agreement was lowest on participant burden and invasiveness (κ = 0.59), the domain that depends most on judging what a new patient would have to do rather than on what the article states. Table S20 reports every figure, and one reviewer had extracted the acquisition protocol for all 31 studies beforehand, so this measures agreement on applying the rubric to a common description rather than on the whole rating pipeline.

To compare diagnostic performance with practical clinical use, we mapped each modality by median reported accuracy, external-validation frequency, median subject-level sample size, and median study-level clinical deployment burden (Figure 2). Using modality means rather than medians yielded the same descriptive burden band for seven of nine modalities. Motor/handwriting shifted from moderate by the median (2.2) to low by the mean (1.96), whereas cognitive/clinical shifted from low by the median (2.0) to moderate by the mean (2.07); both changes occurred near the 2.0 cut point. Omitting each burden domain in turn and recomputing the study-level score from the remaining four left the ordering of the modalities intact, with no modality median moving by more than 0.55; three modalities crossed a descriptive band boundary, in each case from a median already at or beside a cut point (Table S13).

### 3.4. Indicators of Optimism

Three descriptive analyses were consistent with optimistic performance estimates (Figure 3). First, reported accuracy showed a similar distribution for studies with and without external evaluation (Figure 3A), but the externally evaluated subset reporting an accuracy was small (k = 19, against 233 studies without external evaluation), so the within-study internal-to-external changes in Table 3 are more informative than the difference between these distributions. Second, accuracy decreased as total sample size increased (Spearman ρ = −0.22, *n* = 209; Figure 3B). Because this association is between studies and not within them, we examined it in strata and with adjustment (Table S17). The association varied across modalities, from ρ = −0.39 in EEG to ρ = +0.18 in neuroimaging, and pooling within-modality correlations weakened it to ρ = −0.14. In a rank-based model with modality, validation design, dataset type, and external validation as covariates, the association persisted but stayed weak, and the full model explained 13% of the variation in reported accuracy. It was concentrated in studies whose primary evaluation was internal cross-validation (ρ = −0.38) and absent in studies using a single hold-out split (ρ = −0.01), a statistically supported difference in slope (interaction *p* = 0.02). Third, a small number of datasets were reused repeatedly (Figure 3C and Table S10). Counting the datasets studies name rather than the single source category recorded for each, PPMI was named by 46 of the 264 included studies, Kaggle collections by 27, the PhysioNet gait database by 26, and the UCI voice datasets by 21. Because one study may draw on more than one dataset, these counts overlap and do not partition the included set; Table S9 gives the single primary data-source category recorded for each study.

## 4. Discussion

### 4.1. Principal Findings

This systematic review of a modality-stratified sample of 264 machine learning studies on Parkinson’s disease biomarkers found a consistent pattern. Reported diagnostic performance was high across all nine modalities: among the 252 studies reporting accuracy, the median best-model classification accuracy was 95.3%, and modality medians ranged from 91.2% to 97.1%. However, methodological limitations and limited evidence of generalizability were widespread. Under our adapted PROBAST rules, 259 studies (98.1%) were at high risk of bias, and none was at low risk; with the external-validation item excluded and overall risk recomputed across all four domains, 215 (81.4%) remained at high risk on methodological or reporting grounds, and 49 (18.6%) became unclear. Separately, only 20 studies (7.6%) used an independent external cohort. Thus, the main finding is not that one biomarker modality is clearly superior. Instead, most reported accuracies come from internally validated models, and their performance in independent clinical populations remains uncertain. The evidence also addresses a narrower clinical question than this literature framing often suggests. Of the 264 included studies, 251 addressed diagnosis or detection, six severity or staging, three progression or prediction, and four were not classifiable; in other words, almost all discriminate people already carrying a PD diagnosis from comparators. None of the summaries reported here therefore provide evidence on prodromal or preclinical detection, or on predicting who will develop PD. Pre-diagnostic populations are mentioned somewhere in the text of 84 of the 264 reports, most often as motivation in the introduction rather than as the population studied (Table S18), which helps explain why the distinction is easy to lose. Compared with earlier reviews, this review includes studies published through March 2026 and applies PROBAST across all included studies. It also directly compares the practical burden associated with different biomarker modalities.

### 4.2. Reported Performance and Clinical Translation

Reported accuracy clustered in a narrow and very high range, but this should be interpreted with caution. It is unlikely that voice recordings, gait sensors, EEG, MRI, handwriting, and blood biomarkers all provide near-perfect discrimination in independent clinical settings. A more likely explanation is that similar methodological problems inflate performance across modalities. Three patterns were common: evaluation on the same dataset used for development, reuse of a small number of public benchmarks, and reporting of the single best configuration among many. As a result, a clear gap remains between reported model accuracy and clinical use: several years of high-accuracy publications have produced very few externally validated diagnostic tools. Headline accuracy alone therefore gives a misleading picture of the field’s clinical maturity.

### 4.3. The External-Validation Gap

The lack of external validation was the most common weakness, affecting more than nine in ten studies. Of the 20 studies meeting the review’s external-validation definition, 10 provided a directly comparable internal and external figure: performance fell in eight and rose in two. The remaining 10 did not permit a like-for-like comparison because they lacked a distinct internal figure, a comparable external metric, or a comparable case mix. External validation was therefore rare, and where it was done, the results were mixed rather than uniformly poor. Examples include an EEG model that increased from 99.7% to 99.9% [91], a multimodal model that retained 96.2% on the external OASIS-3 cohort [30], a fluid-biomarker model whose AUC fell from 0.83 to 0.72 on an external University of Pennsylvania cohort [21], and a voice model whose AUROC fell from 0.98 to 0.78 under cross-corpus testing [225]. The clearest evidence of optimistic bias came from information leakage in internally validated studies: a neuroimaging methods critique directly demonstrated that slice-level data partitioning inflated accuracy by roughly 26 percentage points, with subject-level accuracy falling from near 100% to about 67% [295]. This type of leakage was demonstrated directly in that study and is likely in others that split recording- or image-level data without clear subject separation, although it was not measured in every study. Even when performance was retained externally, several studies used small, single-institution test sets (e.g., 42 and 73 scans; [54,57]), which is encouraging but may still reflect shared acquisition characteristics and does not establish broad multisite generalizability. Until prospective, multisite external validation becomes routine, reported accuracies should generally be regarded as potentially optimistic estimates rather than as expected clinical performance.

Table 3 classifies the outcome for all 20 studies; only a few allow within-study numerical comparison. Of the 20, ten permitted a directly interpretable internal-to-external numerical comparison: six reported accuracy, three reported AUC/AUROC, and one reported balanced accuracy. Among the six accuracy comparisons, the external figure was lower in four and higher in two, with changes ranging from −29.0 to +1.8 percentage points. All three AUC/AUROC comparisons decreased by 0.11, 0.20, and approximately 0.07–0.12, respectively. Balanced accuracy in the remaining study decreased from approximately 82% to 38–65%. Across the ten comparisons, performance therefore decreased in eight and increased in two. The single largest fall, 29 percentage points, came from a neuroimaging model tested on scans from another dataset. Of the remaining ten studies, eight reported an external evaluation without a comparable internal figure, and two reported non-comparable figures: one because its independent set contained PD participants only, and one because it reported no internal figure distinct from the external one; both are identified in Table S7. These counts are too small to support a pooled estimate of expected degradation, and the studies reporting both figures are self-selected, so the direction of change is more informative than its size. A more defensible conclusion is that, for half of the externally validated studies, the literature does not permit even a within-study assessment of how much performance is lost outside the development data.

Two contributions to the risk-of-bias result should be read separately because they carry different implications. Excluding the external-validation item from the analysis-domain rule and recomputing overall risk across all four domains leaves 215 studies (81.4%) at high risk of bias rather than 259 (98.1%) (Section 3.2). The overall 98.1% estimate should therefore not be interpreted as meaning that 98% of this literature was poorly conducted. Roughly four in five studies remain high risk on methodological or reporting grounds independent of the external-validation item, most often because the sample was inadequate for the model fitted; the remainder reach that rating because they present no evidence that the model works outside its development data. The absence of evidence for generalizability and demonstrated methodological flaws are different findings, and conflating them would overstate the first and understate the second.

### 4.4. Sample Size, Dataset Reuse, and Indicators of Optimism

The three descriptive analyses in Figure 3 provide complementary evidence that the very high reported accuracies should not be interpreted at face value. First, studies with and without external evaluation showed similarly high distributions of their primary best-reported accuracy. This does not indicate that external validation has no effect, because the plotted value is not necessarily external-test performance; within-study comparisons in Table 3 instead show that performance decreased in eight of the ten externally validated studies that permitted direct comparison. Second, reported accuracy showed a modest inverse association with sample size (Spearman ρ = −0.22, *n* = 209), with higher accuracies occurring more often in smaller studies. We do not interpret this as evidence that sample size explains reduced accuracy. This is an ecological, study-level association: the unit is the published study, not the participant, and modality, validation design, dataset type, and task difficulty all vary with sample size. The stratified and adjusted analyses in Table S17 make that concrete. The association is heterogeneous across modalities and is reduced by pooling within them; it survives adjustment for modality, validation design, dataset type, and external validation but remains weak, with the full model accounting for 13% of the variation in reported accuracy. Its clearest structure is in how studies were evaluated: it appears in studies relying on internal cross-validation and is absent in studies using a single hold-out split. This pattern is expected if small cross-validated samples yield optimistically biased estimates rather than genuinely better models, and it is consistent with the simulation evidence on cross-validation in small samples cited in Section 2.6. It remains an observational comparison between groups of studies, so we treat it as one indicator among several rather than as a causal account. Third, a small number of public datasets were reused extensively across the literature, particularly PPMI, Kaggle collections, PhysioNet gait data, and UCI voice datasets. Repeated analysis of the same benchmark populations can create an appearance of replication without demonstrating generalization to independent clinical populations. Taken together, these patterns support cautious interpretation of near-perfect internal accuracies rather than treating them as estimates of expected clinical performance. A fourth mechanism acts on the metric itself. Raw accuracy is the figure this literature reports most consistently, and it is the figure least robust to unequal class sizes: a model that predicts the majority class for everyone can post a high accuracy while identifying few members of the minority class, and in case-control designs with unequal groups, in multi-class staging tasks, and in cohorts assembled around a single diagnosis, this is a live possibility rather than a theoretical one. We cannot quantify its contribution here, because the number of outcome classes and the class distribution of each test set were not extracted and cannot be recovered reliably from the reports (Section 3.3, Table S18), and because the alternatives are not interchangeable: balanced accuracy and the F1 score behave differently under imbalance, and F1 depends on which class is treated as positive and on how it is averaged across classes. What follows is a reporting recommendation rather than a correction we can apply: studies should state the class counts of the evaluation set, report class-specific sensitivity and specificity or a confusion matrix, and, where a summary figure is given, define its averaging explicitly.

Repeated use of benchmark datasets also raises concern about subject-level information leakage because many repositories contain multiple recordings, images, or signals from the same participant. If observations from one participant appear in both the training and test sets, model performance can be substantially inflated. In 46 studies, only recording-, image-, or signal-level counts were reported, so we could not establish an interpretable subject-level sample size. Near-100% accuracies in studies based on small handwriting, EEG, gait, or imaging datasets therefore warrant particular caution when subject-independent partitioning is not clearly documented.

### 4.5. Accuracy and Deployment Burden Across Modalities

Because reported accuracy differed relatively little among modalities, practical burden provides another useful way to distinguish them. Figure 2 shows a much wider separation in deployment burden than in reported accuracy. Voice/speech had the lowest median burden (1.0), followed by motor/handwriting (2.2), while EEG, fluid/molecular biomarkers, and neuroimaging had progressively higher values of 3.4, 4.0, and 4.2. Cognitive/clinical was also low (2.0), although only three studies were included in that category.

Multimodal/fusion is particularly informative. Its median burden was 2.6, but scores ranged widely (IQR 1.6–4.2). Combining modalities therefore does not necessarily imply a high-burden test; the burden depends mainly on the data being combined. Voice, video, or simple wearable measurements can remain relatively easy to collect, whereas combinations involving MRI, molecular assays, or other specialized procedures are considerably more demanding.

Lower deployment burden should not be interpreted as stronger clinical evidence. Voice studies, for example, have low acquisition burden but frequently rely on reused public datasets and still require stronger external validation. Neuroimaging requires greater infrastructure, while fluid biomarkers add biospecimen collection and laboratory processing. These differences describe acquisition and implementation requirements, not validated clinical roles. Whether any modality is suitable for screening, diagnosis, or confirmation must be established prospectively in the intended target population [30,57,141,225].

It is useful to separate three questions often combined under the term “clinical readiness.” The first is internal technical performance: whether a model discriminates well in its development setting. The second is generalizability: whether performance is retained in independent data from another site, cohort, or language. The third is clinical implementation: whether the test can be delivered to the intended population at acceptable cost, burden, and risk and whether it improves decision-making. This review summarizes the first and, for a minority of studies, the second; it does not evaluate the third, and no included study reported a prospective clinical evaluation. By this framework, most of the literature addresses only the first question. This is more precise than simply describing the field as not clinically ready because it identifies the specific missing evidence.

Translation readiness should not be inferred from accuracy or deployment burden alone. The burden score describes practical implementation burden, whereas the risk-of-bias assessment addresses the credibility and generalizability of the reported performance. A modality may be easy to scale in practice yet lack evidence of generalizability, while a more burdensome modality may have stronger validation evidence.

### 4.6. Emerging Methodological Directions

Two methodological trends may help improve generalization. First, self-supervised and foundation-model speech embeddings (e.g., wav2vec and WavLM representations) are increasingly used instead of handcrafted acoustic features, and at least one such study validated a fusion model across an external clinical cohort [222], suggesting that transfer learning from large unlabeled datasets may improve generalization, not only accuracy. Second, multimodal fusion is increasingly common; however, in this corpus, fusion did not consistently outperform the best single-modality models, and its added pipeline complexity was rarely matched by stronger validation. Multimodal fusion is most useful when modalities provide complementary information and when external validation supports the added complexity. Explainable AI methods were reported more often but applied inconsistently and cannot replace rigorous validation.

### 4.7. Implications for Practice and Reporting

These findings identify several reporting priorities for future PD machine learning studies. Report subject-level sample sizes and subject-independent data partitioning clearly, and separate model selection from final evaluation. Independent external testing and, ideally, prospective multisite validation should support claims of generalizability. The PD diagnostic reference standard should also be stated explicitly; 61.0% of studies did not report how PD diagnosis was established. Finally, reporting all tested models rather than only the best-performing configuration would provide a less selective picture of performance. Table S11 maps these recommendations to TRIPOD+AI [296] and PROBAST+AI [297].

### 4.8. Strengths and Limitations

Strengths of this review include its broad coverage of 264 studies across nine modalities, high agreement in the double-screened subsample (Cohen’s κ = 0.862, *n* = 147), human eligibility assessment of every retrieved report, human verification of the extracted data and risk-of-bias judgments for all included studies, and use of PROBAST. The review also has several limitations. The search covered January 2020 to March 2026; eligibility confirmation identified nine reports published before the window, three first available after the cutoff, one with self-reported PD status, and seventeen without a PD-versus-comparator diagnostic classification used in this review (Note S1). Because modalities, algorithms, datasets, and reporting differed substantially, we used a narrative synthesis rather than a meta-analysis. We extracted each study’s best-performing configuration as the primary result; although this follows common practice, it may increase the optimistic bias discussed above. Because we did not extract performance values for the configurations we did not select, we could not test an alternative model-selection rule, so the medians reported here describe best-model performance rather than the performance expected from a model chosen without reference to its result. Some studies reported metrics incompletely, and modality classification required judgment when datasets included multiple data types. The number of outcome classes was not extracted either, and we show in Section 3.3 that it cannot be recovered reliably from the full texts, so differences in task difficulty remain an unmeasured source of heterogeneity in every performance summary we report. Raw accuracy is also the metric this literature reports most consistently, and it is the metric least robust to class imbalance; because test-set class distributions were not extracted, we cannot estimate how much of the reported performance it flatters. The sample-size item was re-rated during revision under a rule frozen before the source recheck (Section 2.6). This is a post hoc change from the numeric bands used at submission, recorded as a protocol deviation, and the 500-participant boundary for end-to-end deep models is a review-specific choice rather than a validated threshold; it accounts for most of the difference from the earlier judgments, and the alternative boundaries are reported in Table S6. Because we did not retain the reasons for individual judgments made before revision as a structured field, we cannot reconcile the two sets of answers study by study beyond the comparison in Table S6. The clinical deployment-burden rubric was developed for this review rather than externally validated and necessarily involves judgment; therefore, we interpret it descriptively, with full scoring anchors, per-study ratings, and source-based rationales provided in Supplementary Tables S13 and S14. The five domains also enter the composite unweighted, which asserts that they matter equally and is a simplifying choice rather than a finding; the leave-one-domain-out analysis in Table S13 shows which modality positions depend on it. A defined route to validation would make the rubric more useful than the descriptive instrument it currently is. We suggest three steps: a Delphi process among clinicians, people living with Parkinson’s disease, and implementation and health-service stakeholders, to agree on the domains and the weights they should carry; an independent-rater reproducibility study of the agreed instrument; and comparison of its scores against observed acquisition costs, staff time and facility requirements in real services. Consensus alone would establish content and weighting, not predictive or criterion validity, which is why the third step matters.

Several additional limitations should be considered. The principal reduction without human full-text assessment was the modality-stratified sampling of the 2046 records retained after title-and-abstract screening to 377 records; hand-searching added two reports, giving 379 reports sought for retrieval (Section 2.4). A reviewer assessed every available full-text file against the eligibility criteria; Section 2.4 describes the single mismatched-file case and its subsequent E6 exclusion. The included set is therefore a purposively sampled subset of the retained literature rather than an exhaustive census; modality counts reflect the sampling procedure, and the proportions reported here describe the studies assessed rather than the field as a whole. An audit of the 1669 records not selected (Section 3.1 and Table S16) found similar abstract-reported accuracy and similar use of external-validation language, which argues against a large distortion of the performance findings. Because abstracts cannot support a PROBAST assessment, however, whether the risk-of-bias proportions generalize to the unselected literature remains untested. Each of the 264 included studies therefore carries a single human risk-of-bias rating rather than two (Section 2.6), and Table S5 reports machine-versus-final concordance for that stage. Reproducibility was instead measured on a subset: both reviewers independently rated the same 31 studies, and their ratings agreed on the overall judgment for 30 of them (Section 3.2, Table S19). Two limits of that exercise should be kept in view. It measured how consistently the two of us applied the tool, not whether our shared reading was correct, so a systematic misreading common to both would not show up. And because it rested on 31 studies in which the overall rating was almost invariant, its precision on the overall rating was limited, which is why we report agreement at the item and domain level as well. Separately, the analysis-domain sample-size item was re-rated during revision under the frozen rule of Section 2.6, with a reviewer reading the source article for 103 of the 264 studies and the remaining 161 resting on the rule applied to automatically extracted inputs (Table S6). Single verification also leaves the recorded ratings open to reviewer-specific interpretation, and to anchoring on the automated pre-assessment the reviewer was checking rather than producing from scratch; we cannot establish the direction or size of either effect from these data. Inter-reviewer agreement is available for title-and-abstract screening, where 147 records were double-screened (Cohen’s κ = 0.862); the remaining records were single-screened or assigned by classifier. Because the reviewers verified disjoint modality groups, differences between their verification sets cannot be separated from genuine differences among those groups.

The review was further limited to English-language reports and to three bibliographic databases. Scopus and the ACM Digital Library were unavailable through our institutional access, and we could not search Google Scholar systematically because automated queries were blocked. These restrictions may have introduced language and database-coverage bias. Formal publication-bias tests are not well established for heterogeneous diagnostic-accuracy outcomes, although the negative relationship between accuracy and sample size (Figure 3B) is consistent with a small-study effect. Very high accuracies from small or poorly documented public datasets (Table S12) should therefore be treated as cautionary examples rather than benchmarks. Finally, 177 of 264 studies reported a PD-group size that could be interpreted at the participant level (median 53, range 5–2083), and 24 enrolled fewer than 20 people with PD. The protocol required at least 20 participants in total, not 20 participants with PD, so an eligible study could still include a very small PD group.

## 5. Conclusions

Recent machine learning literature on Parkinson’s disease reports high accuracy across all biomarker modalities. The central finding of this review is that reported performance has matured much faster than the evidence that these models generalize beyond the data they were built on. Of 264 studies, 98.1% were at high overall risk of bias under our adapted PROBAST rules, and 81.4% remained at high overall risk after excluding the external-validation item and recomputing across all four domains, while only 7.6% used independent external validation. Reported accuracy should therefore be interpreted with caution, especially when studies rely on internal validation, repeated use of public datasets, or data partitioning that may allow information leakage. The field does not primarily need another model reporting 98–99% internal accuracy; it needs evidence that existing models generalize. Among the 20 studies that evaluated externally, only ten reported figures allowing a within-study comparison, so for most of this literature, the size of the loss on new data is simply unknown. These proportions describe the studies assessed here, which were drawn by modality-stratified sampling rather than as a census of the field. Closing this gap requires subject-independent testing and external, preferably multisite, validation. Studies also need to clearly report participant-level sample sizes, diagnostic criteria, and the model-selection process. From an acquisition-burden perspective, voice and wearable sensing were less demanding to acquire, whereas neuroimaging and fluid biomarkers required greater acquisition and infrastructure. These differences describe implementation burden only and do not establish screening, diagnostic, or confirmatory roles. Almost every included study classified participants with established PD; clinical utility must therefore be established prospectively in the relevant target population.

## Figures and Tables

**Figure 1:**
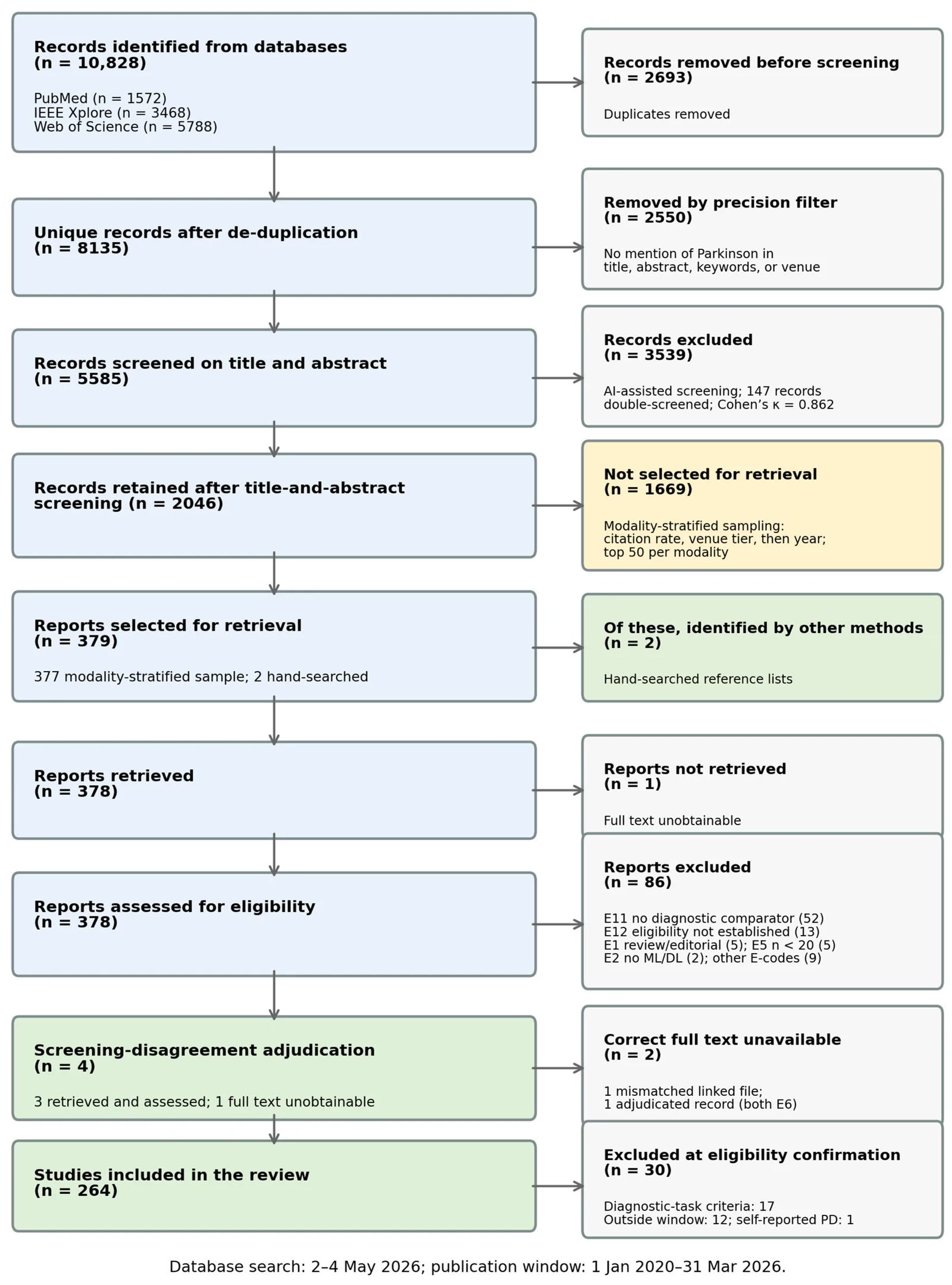
PRISMA 2020 flow diagram for study selection.

**Figure 2:**
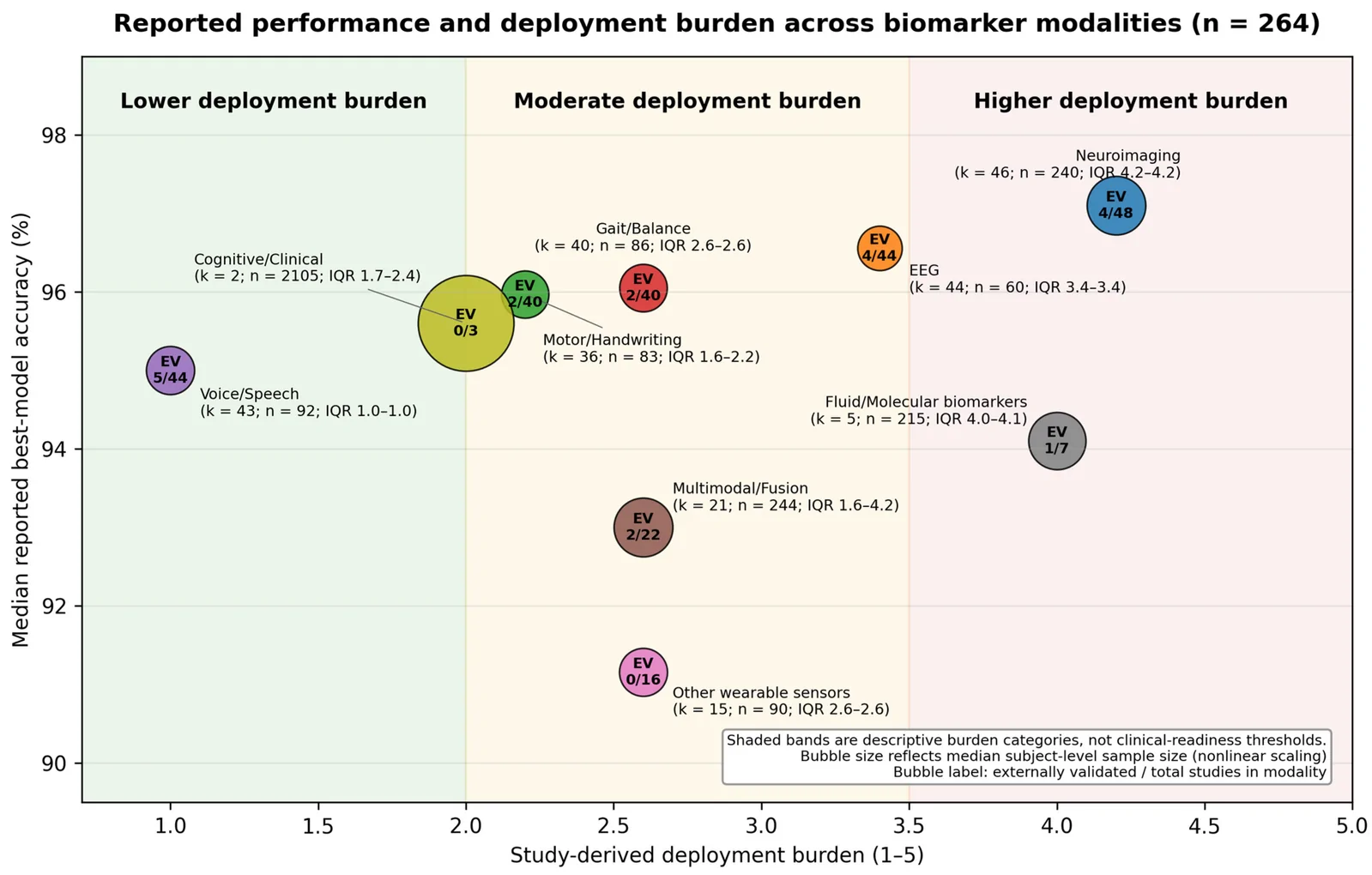
Reported performance and deployment burden across Parkinson’s disease biomarker modalities (*n* = 264). Each bubble is one modality, positioned by median reported best-model accuracy and the median study-level clinical deployment-burden score. For each included study, acquisition cost, participant burden/invasiveness, equipment/infrastructure requirements, scalability/throughput, and remote-collection feasibility were rated from 1 (lowest burden) to 5 (highest burden) and averaged without weighting; the x-coordinate is the median of those study-level scores within the modality. Bubble size reflects the median subject-level sample size using nonlinear scaling for readability; the bubble center shows the number of externally validated studies relative to the total, and the label reports the number contributing an accuracy value (k), median subject-level *n*, and burden IQR. Shaded bands are predefined descriptive categories: low burden ≤ 2.0, moderate burden > 2.0 and <3.5, and high burden ≥ 3.5. The review-specific rubric is not a validated clinical scale. Supplementary Tables S13 and S14 provide the scoring anchors, decision rules, modality summaries, and all study-level ratings.

**Figure 3:**
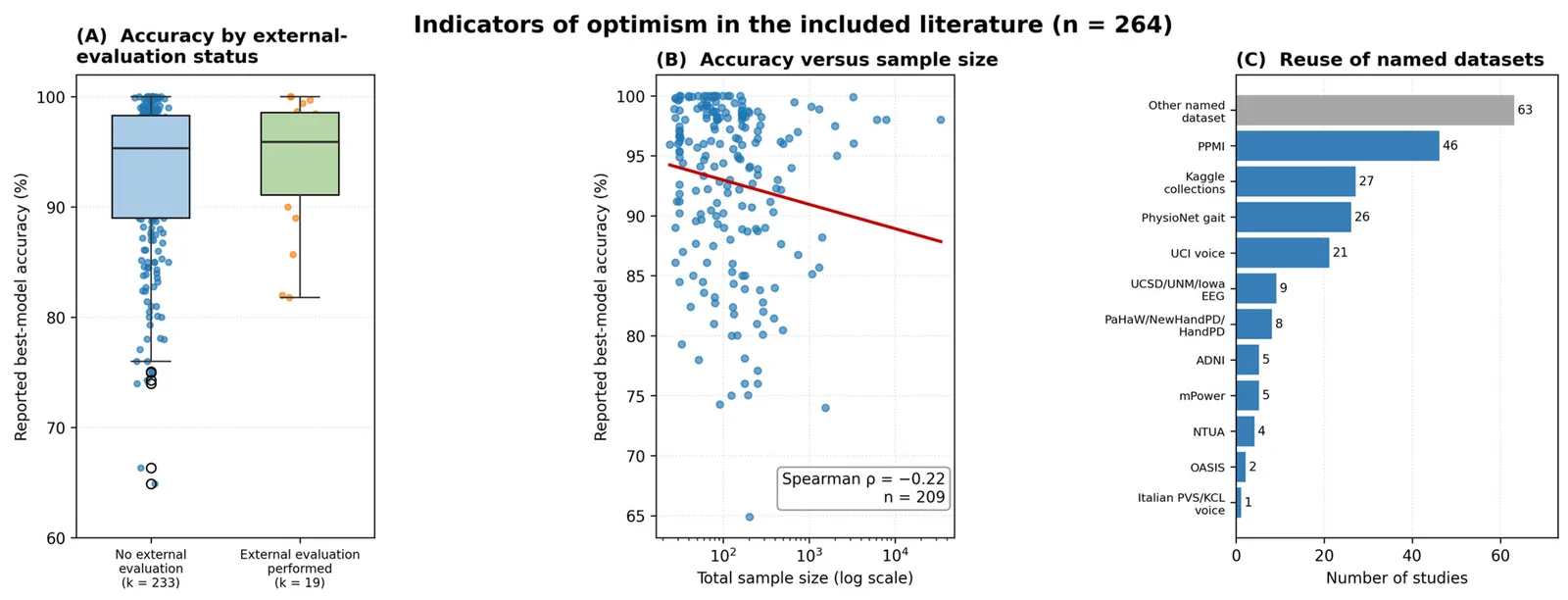
Indicators of optimism in the included literature (*n* = 264). (**A**) Primary best reported accuracy among the 252 studies reporting accuracy, grouped by external-validation status: 19 of the 20 externally validated studies and 233 of the 244 studies without external validation reported an accuracy value. The plotted value is each study’s best reported accuracy and should not be interpreted as external-test-set accuracy; the within-study internal-to-external changes in Table 3 are more informative. (**B**) Best reported accuracy against total sample size on a log scale for the 209 studies reporting both. The red line shows the ordinary least-squares linear trend fitted to accuracy as a function of log10-transformed total sample size; Spearman ρ = −0.22 (*n* = 209), indicating that smaller studies more often reported higher accuracies. (**C**) Number of studies drawing on each named dataset family, showing the concentration of the literature on a small number of public collections.

**Table 1:** Risk-of-bias ratings ^1^ across the four PROBAST domains (*n* = 264).

Domain	Low Risk, *n* (%)	Unclear, *n* (%)	High Risk, *n* (%)	Key Concern
D1 Participant selection	151 (57.2)	65 (24.6)	48 (18.2)	Convenience/public-dataset sampling
D2 Predictors/index test	98 (37.1)	158 (59.8)	8 (3.0)	Incomplete reporting of ML pipeline
D3 Outcome/reference standard	37 (14.0)	215 (81.4)	12 (4.5)	PD diagnostic criteria not stated (61.0%)
D4 Analysis	0 (0.0)	5 (1.9)	259 (98.1)	No external validation; small samples
Overall	0 (0.0)	5 (1.9)	259 (98.1)	—

^1^ Overall risk of bias was rated high if any domain was high, unclear if any domain was unclear without a high rating, and low only if all domains were low. The analysis domain includes the external-validation signaling question, which 232 of the 264 studies failed; with that item excluded and overall risk recomputed across all four domains, 215 studies (81.4%) remain at high risk and 49 (18.6%) become unclear (Section 3.2). Methodological risk of bias and absence of evidence for external generalizability are therefore reported as separate findings.

**Table 2:** Diagnostic-performance summary by modality (*n* = 264).

Modality	*n*	Median Acc. ^1^ % (Range)	Common Datasets	Dominant Methods	Ext. Val. ^2^	High RoB ^3^
Neuroimaging	48	97.1 (65–100; k = 46)	PPMI	CNN/transfer learning	4	47/48
EEG	44	96.6 (75–100; k = 44)	UC San Diego, UNM	CNN, SVM	4	44/44
Motor/Handwriting	40	96.0 (78–100; k = 36)	Private, NewHandPD	SVM, CNN/ResNet	2	39/40
Gait/Balance	40	96.1 (78–100; k = 40)	PhysioNet, private	SVM, random forest	2	40/40
Voice/Speech	44	95.0 (76–100; k = 43)	UCI Oxford/Sakar	SVM, KNN, CNN	5	43/44
Multimodal/Fusion	22	93.0 (80–100; k = 21)	Private, PPMI	SVM, deep fusion	2	21/22
Other Wearable Sensors	16	91.2 (74–100; k = 15)	Private	SVM, random forest	0	16/16
Fluid/Molecular Biomarkers	7	94.1 (87–100; k = 5)	PPMI	Random forest, DNN	1	6/7
Cognitive/Clinical	3	95.6 (95–96; k = 2)	Mixed	Ensemble/AdaBoost	0	3/3

^1^ “Median acc.” is over studies reporting an accuracy value; ^2^ “Ext. val.” counts studies using an independent external dataset (PROBAST analysis item); ^3^ “High RoB” is the number of studies at overall high risk of bias. k is the number of studies contributing an accuracy value to each median, so medians for small categories (e.g., fluid, k = 5; cognitive/clinical, k = 2) rest on very few studies and should not be over-interpreted. Medians pool classification problems of different difficulty, including multi-class and severity tasks, which the review could not separate (Section 3.3); Supplementary Table S3 gives the per-study detail. Abbreviations: acc., accuracy; Ext. val., external validation; RoB, risk of bias; k, number of studies adding to the statistic. PPMI, Parkinson’s Progression Markers Initiative; UC San Diego, University of California San Diego; UNM, University of New Mexico; UCI, University of California Irvine machine learning repository; NewHandPD, New Handwritten Parkinson’s Disease dataset; CNN, convolutional neural network; SVM, support vector machine; KNN, k-nearest neighbors; DNN, deep neural network.

**Table 3:** The 20 studies meeting the review’s prespecified external-validation definition: internal vs. external performance.

Study	Modality	External Dataset	Internal → External Performance, Where Reported	Outcome
Li [54]	Neuroimaging	Dong-Hai Centre (different scanner)	External AUC 0.957; external accuracy 89%	Retained
Gupta [57]	Neuroimaging	SSH Jammu hospital MRI (trained on PPMI)	Accuracy 96.4% → 98.2%	Retained
Bunterngchit [91]	EEG	Univ. of Iowa EEG	Accuracy 99.7% → 99.9%	Retained
Qiu [99]	EEG	UCSD/UNM/Iowa (cross-dataset)	Accuracy 92.5% → 90.2%	Small drop
Galaz [141]	Motor/Handwriting	Czech/Hungarian/US/Colombian (leave-one-language-out)	Balanced accuracy ~82% → ~38–65%	Substantial drop
Adnan [222]	Voice/Speech	External clinical + unseen English speech	AUROC 0.89 → 0.77–0.82	Moderate drop
Klempír [225]	Voice/Speech	Italian + KCL (leave-group-out)	AUROC 0.98 → 0.78	Substantial drop
Zhi [30]	Multimodal/Fusion	OASIS-3, PDBP	Accuracy 97.5% → 96.2%/95.8%	Retained
Hu [21]	Fluid/Molecular Biomarkers	UPenn cohort	AUC 0.83 → 0.72	Substantial drop
Pareek [226]	Voice/Speech	Lithuanian voice dataset (independent cohort)	External numerical result not reported separately	Not reported
Mukherjee [93]	EEG	Iowa dataset (independent cohort, different site)	Accuracy 99.4% → 95.3%	Drop
Haoyu Wu [128]	EEG	Independent cohort, different site—channels selected on the University of New Mexico data, tested on the University of Iowa data	Internal 100.0%; external not reported separately	Not separately reported
Cankun Zhong [190]	Gait/Balance	Leave-one-dataset-out across the independent Ga, Ju and Si cohorts	Internal 95.9%; external not reported separately	Not separately reported
Xiaotian Wang [207]	Gait/Balance	Cross-dataset held-out Ga/Ju/Si cohort	Internal 98.5%; external not reported separately	Not separately reported
Huimin Lu [150]	Motor/Handwriting	Independent cohort—trained on PaHaW, validated on the Cc-PhD cohort	Internal 98.7%; external not reported separately	Not separately reported
Talitckii [268]	Multimodal/Fusion	Independent cohorts—handwriting experiment trained on HandPD and tested on two datasets not used in training; described by the authors as their second experiment. The headline sensor/video result uses internal CV and is not the external one.	Internal 90.0%; external not reported separately	Not separately reported
Hussain [70]	Neuroimaging	Independent cohort—pretrained model tested on 40 unseen MRI scans from another dataset (20 PD, 20 HC); a secondary generalization experiment, the primary result being a hold-out evaluation.	Internal 96.0% → accuracy 67%, sensitivity 65%, specificity 70%, AUC 0.65 (internal 96%)	Large drop
Zhang [81]	Neuroimaging	Cross-dataset/cross-disorder transfer—model pretrained on ABIDE, ADHD-200, and OASIS-3, then evaluated on PPMI, as reported in Table VI of the source article. This is transfer into PPMI from non-PD cohorts, not validation in a second independent PD cohort.	Internal 81.8% → accuracy 81.8% on the PPMI target set	External figure only
Amato [230]	Voice/Speech	Cross-corpus—a second database was added specifically to run cross-corpus validation (18 PD, 19 HC); primary analysis is a 70/30 speaker-independent hold-out on PC-GITA.	Internal 99.4%; external not reported separately	Not separately reported
Liaqat Ali [251]	Voice/Speech	Independent test set of 28 PD participants (PD-only, so specificity is not estimable on it)	Internal 100.0% → unbiased accuracy 96.42% on the independent set	Small drop

External validation = evaluation on an independent cohort or dataset not used in training (from a different site or dataset); within-study hold-out and cross-validation are not counted. Of the 20 externally validated studies, 10 permitted a directly comparable internal-to-external numerical comparison; performance decreased in eight and increased in two. The remaining 10 did not permit a like-for-like comparison because a distinct internal figure, comparable external metric, or comparable case mix was unavailable. Bracketed numbers refer to the reference list. Abbreviations: AUC/AUROC, area under the receiver operating characteristic curve; UCSD, University of California San Diego; UNM, University of New Mexico; KCL, King’s College London; OASIS-3, Open Access Series of Imaging Studies-3; PDBP, Parkinson’s Disease Biomarkers Program; UPenn, University of Pennsylvania.

## Data Availability

All data analyzed in this review were extracted from previously published studies, which are cited herein. The complete data-extraction table, per-study risk-of-bias ratings, and full search strings are provided in the Supplementary Materials. No new primary data were generated.

## Supplementary Materials

The following supporting information can be downloaded at: https://www.mdpi.com/article/10.3390/brainsci16101027/s1, Table S1, PRISMA 2020 checklist [34]; Table S2, complete search strings; Table S3, complete data extraction and reported performance for all 264 included studies; Table S4, adapted PROBAST signaling questions; Table S5, risk-of-bias concordance (initial automated vs. final human-verified ratings); Table S6, per-study PROBAST risk-of-bias ratings; Table S7, external-validation detail (type, dataset scope/size, internal→external outcome); Table S8, diagnostic performance stratified by task type; Table S9, primary data-source category of each study; Table S10, reuse of named datasets across the 264 studies; Table S11, mapping of reporting recommendations to TRIPOD+AI [296], PROBAST [298], and PROBAST+AI [297]; Table S12, high-risk exemplars; Table S13, modality-level clinical deployment-burden summary, scoring rubric, and sensitivity analyses; Table S14, study-level deployment-burden ratings for all 264 included studies; Table S15, record-level study ledger giving the disposition of every record from retrieval onward; Table S16, selection-bias audit comparing the records selected for full-text assessment with those not selected; Table S17, stratified and adjusted analyses of the association between sample size and reported accuracy; Table S18, recoverability of task structure and of pre-diagnostic populations from the full texts; Table S19, inter-rater agreement between the two reviewers on the 31 independently dual-rated studies; Table S20, inter-rater agreement on the deployment-burden rubric for the same 31 studies; Table S21, the two language-model-assisted steps in the data-extraction and PROBAST pre-assessment pipeline, with their settings and verbatim prompts; Figure S1, PROBAST risk-of-bias summary; Note S1, protocol deviations; Supplementary Code S1, Python script used to generate Figure 1, Figure 2 and Figure 3 and Figure S1; Supplementary Code S2, Python script reproducing the selection-bias audit; Supplementary Code S3, Python scripts reproducing the sample-size rule, its per-study inputs and the sensitivity analyses; Supplementary Code S4, Python scripts reproducing Tables S17 and S18; Supplementary Code S5, Python scripts reproducing Tables S19 and S20; Supplementary Code S6, Python script regenerating Table S21 from the two pipeline scripts, which are supplied with it.
