# Supplementary material for: Machine Learning for Parkinson’s Disease Biomarkers: A Systematic Review of Diagnostic Performance, Risk of Bias, and Clinical Translation Readiness
Source: Brain Sci. 2026 Sep 26;16(10):1027. doi: 10.3390/brainsci16101027 (PMC13702696; doi:10.3390/brainsci16101027)
Supplement: Supplementary file 1 [file brainsci-16-01027-s001.zip › brainsci-4564970-supplementary/Figure_S1_PROBAST_Risk_of_Bias.pdf]

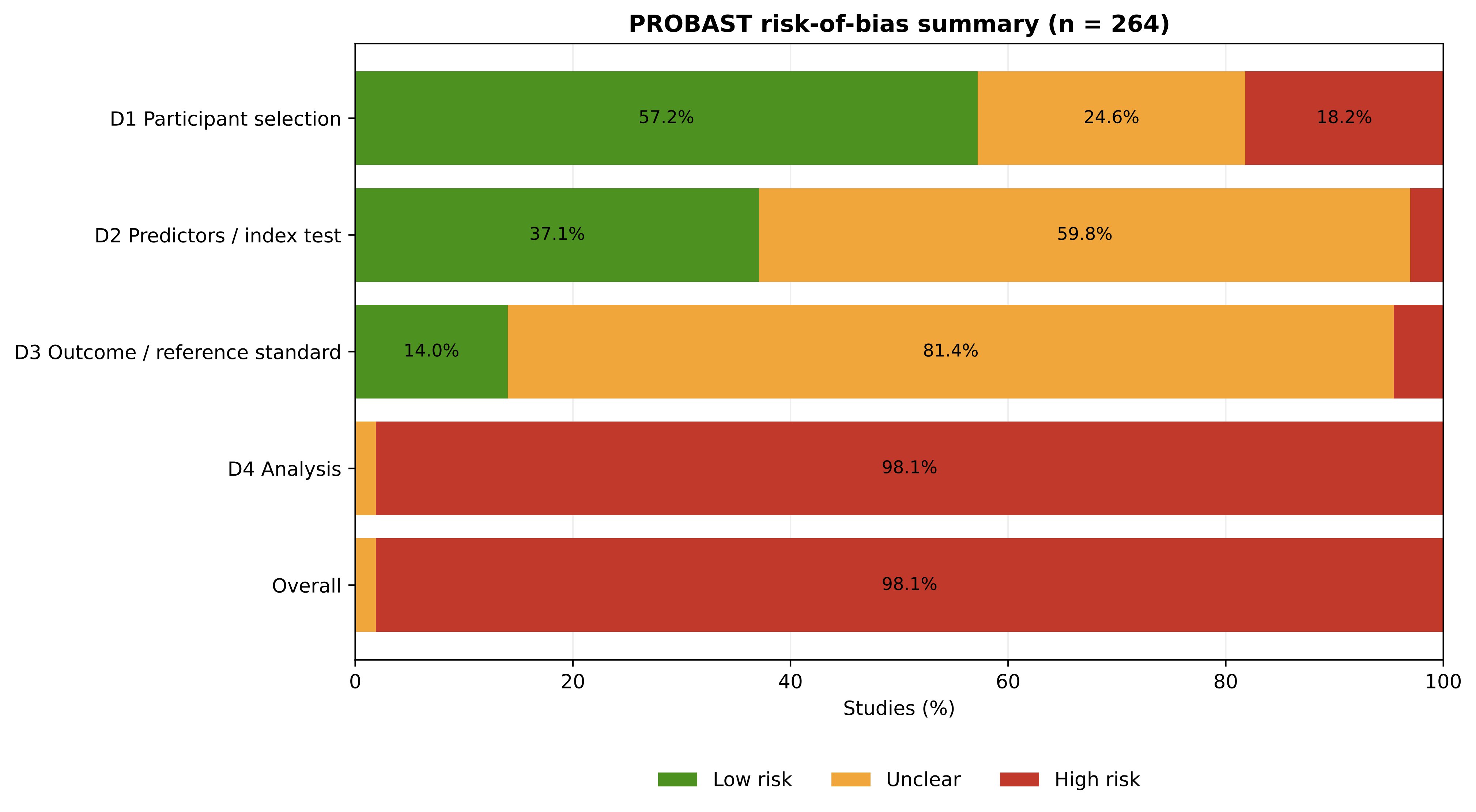

PROBAST risk-of-bias summary (n = 264)
D1 Participant selection
D2 Predictors / index test
D3 Outcome / reference standard
D4 Analysis
Overall
57.2%
24.6%
18.2%
37.1%
59.8%
14.0%
81.4%
98.1%
98.1%
0
20
40
60
80
100
Studies (%)
Low risk
Unclear
High risk
